# A Regulatory Loop Involving Notch and Wnt Signaling Maintains Leukemia Stem Cells in T-Cell Acute Lymphoblastic Leukemia

**DOI:** 10.3389/fcell.2021.678544

**Published:** 2021-06-11

**Authors:** Ling Zhang, Jieying Wu, Yashu Feng, Bijay Khadka, Zhigang Fang, Jiaming Gu, Baoqiang Tang, Ruozhi Xiao, Guangjin Pan, Jia-Jun Liu

**Affiliations:** ^1^Department of Hematology and Hematology Institute of Sun Yat-sen University, The Third Affiliated Hospital of Sun Yat-sen University, Guangzhou, China; ^2^Guangzhou Institutes of Biomedicine and Health, Chinese Academy of Sciences, Guangzhou, China

**Keywords:** T-cell acute lymphoblastic leukemia, Wnt, notch, cancer stem cells, relapse

## Abstract

Leukemia-initiating cells play critical role in relapse, resistance to therapies and metastases but the mechanism remains largely elusive. We report that β-catenin is over-expressed in almost all T-ALL patients and flow sorted β-catenin^high^ fractions are highly resistant to therapy, leading to liver metastases in nude mice as well as dysregulated lncRNAs. Pharmacological inhibition through XAV-939 as well as si-RNA mediated inhibition of β-catenin is initially effective in re-sensitization to therapy, however, prolonged inhibition shifts dependency from β-catenin to Notch signaling, with particularly high levels of receptors Notch 1 and Notch 2. The results are verifiable in a cohort of T-ALL patients comprising of responders vs. those who have progressed, with β-catenin, Notch 1 and Notch 2 elevated in progressed patients. Further, in patients-derived cells, silencing of Notch 1 or Notch 2 does not counter resistance to β-catenin inhibition, rather pharmacological pan-Notch inhibition is needed to overcome resistance and its effect on *in vitro* tumor sphere formations as well as *in vivo* liver metastases. Thus, wnt and Notch signaling are part of a regulatory loop mutually compensating for each other in T-ALL, while ensuring the maintenance of stem cell phenotype.

## Introduction

T-cell acute lymphoblastic leukemia (T-ALL) is aggressive, as its name suggests, progresses rather quickly. The T-cell variant of ALL accounts for about 15% ALLs in children and about 25% of ALLs in adults (Chiaretti and Foa, 2009; Marks and Rowntree, 2017). Although largely curable, with almost 50% survival after 5 years, the prognosis is rather poor in patients with relapsed disease (Marks and Rowntree, 2017). This calls for better understanding of factors that can potentially lead to T-ALL relapse and progression. Cancer stem cells and the process of “stemness” have long been associated with cancer relapse (Ahmad, 2013; Suresh et al., 2016; Peitzsch et al., 2017; Khandekar et al., 2019; Yin et al., 2021). This is even true for T-ALL with the realization of existence as well as a role of stem cells in relapse, drug resistance and metastasis (Tremblay and Curtis, 2014; Tan et al., 2017), however, the mechanistic details remain largely unexplored.

Giambra et al. (2015) established that T-ALL subpopulations with active wnt signaling are enriched with leukemia-initiating cells, thus establishing a connection between wnt signaling and the cancer stem cell phenotype in T-ALL. Stemness in T-ALL possibly has epigenetic basis as well (Zhu et al., 2018), however, the epigenetic regulation involving Spi1 also needs β-catenin, a component of wnt signaling pathway (Zhu et al., 2018). These findings suggest an essential role of wnt signaling in establishing and/or maintaining the stemness in T-ALL. β-catenin also plays critical role in proliferation and survival of leukemia (Chung et al., 2002) and, thus, offers as an attractive target for therapy.

The goal of this study is to mechanistically dissect the role of β-catenin in stemness of T-ALL, particularly in patient-derived samples. We evaluated the role of β-catenin in inducing drug resistance and metastasis of T-ALL. Further, in view of the relapse observed in clinics, we mimicked such conditions to evaluate alternate signaling pathways that can compensate for the effective targeting of β-catenin. Our results indicate an intricate relationship between wnt and Notch signaling that helps maintain the stemness in T-ALL patients.

## Materials and Methods

### Patients

The study involving human subjects was approved by the Institutional Review Board and the Ethics Committee at Sun Yat-sen University. A signed consent form was received from all patients and volunteers. The identifying information for T-ALL patients as well as healthy volunteers was not revealed to the researchers.

### Drug Treatments

Cells were treated with VDL (V: 0.005 μM vincristine; D: 0.05 nM dexamethasone and L: 0.0025 IU L-asparaginase) for 10 days. Control cells were left untreated. Cells were then harvested by centrifugation at 500 rcf for 5 min and re-suspended in DPBS (Dulbecco’s phosphate-buffered saline, Invitrogen) containing FBS at a final concentration of 2.5%. XAV-939 and BMS906024 used in the study were procured from Sigma (China).

### ELISA Assays

Active Beta Catenin Pathway Assay Kit (Novus Biologicals) was used to quantitate β-catenin in patient-derived samples, following manufacturer’s suggested protocol. This qualitative ELISA kit is specifically designed to detect the active dephosphorylated form of β-Catenin. Notch family receptors were quantitated using specific ELISA kits for Notch1, Notch2, Notch3 and Notch4 (Seajet Scientific, Shanghai, China—LS Bio—LifeSpan Biosciences). C-myc and Spi1 were also quantitated using specific ELISA kits purchased from Seajet Scientific, Shanghai, China.

### Flow Cytometry and Cell Sorting

Single cell suspensions, made using GentleMACS kit (Miltenyi Biotec, China), from patient samples were incubated in phosphate-buffered saline containing 1% FBS with human beta-Catenin APC-conjugated Antibody (R&D Systems, China), and isotype-matched mouse immunoglobulins were used as controls. Samples were analyzed and sorted, using an EPICS ALTRA flow cytometer (Beckman Coulter, China). For the positive and negative population, only the top 10% most brightly stained cells or the bottom 10% most dimly stained cells were selected, respectively.

### Animal Studies

Our protocol for *in vivo* experiments, involving NSG mice (Vital River Laboratories, Co., Ltd., China), was approved by the Animal Research Ethics Committee at the Sun Yat-sen University. All methods were performed in accordance with the relevant guidelines and regulations. All mice were housed in sterilized animal facility. Based on our initial titrations, we injected 50,000 cells into the tail veins of NSG mice and euthanized mice after 7 weeks. The livers were visually inspected for signs of metastases.

### Immunohistochemistry

Samples from hepatic metastases were sectioned, conventionally stained with HE and immunohistochemically labeled with anti-cytokeratin using R-IHC and standard IHC and examined by a trained pathologist.

### Trypan Blue Assay

Trypan blue dye exclusion assay was used to evaluate cell viability. Trypan blue (Sigma, China) (0.4%) was added in 1:1 ratio with the single cell suspensions and the cells monitored and quantitated under bright field microscope. The live cells did not retain blue stain and their cytoplasm was clear while the dead cells were marked with blue cytoplasm.

### Tumorospheres

First, single cell suspensions were ensured enzymatically using trypsin and mechanically using a 22 gauge needle for 2 min. For the generation of tumorospheres, cells were plated in 24-well ultra-low attachment plates (Corning Inc., China) at a density of 5,000 viable cells/well. Average sphere forming efficiency was evaluated after 18–21 days of culture under an inverted Olympus IX70 microscope camera (Olympus, China) by counting spheres that were larger than 50 μm in diameter using the ImageJ Software.

### Quantitative RT-PCR

Trizol reagent (Life Technologies, China) was used to isolate RNA following the manufacturer’s protocol. Quantitative RT-PCR reactions were performed on an ABI 7500 RT-PCR system (Applied Biosystems) using validated primers from BioRad (China). Only RNAse-free water was used throughout the assays.

### Statistics

All experiments were performed in triplicates. Two-tailed independent Student’s *t*-test and ANOVA were used to compare tested groups. Differences were considered significant when *p* < 0.05. Statistical analyses were conducted using Graphpad Prism software.

## Results

### β-Catenin Expression in T-ALL Patients and Hepatic Metastasis of β-Catenin Expressing Cells

**FIGURE 1:**
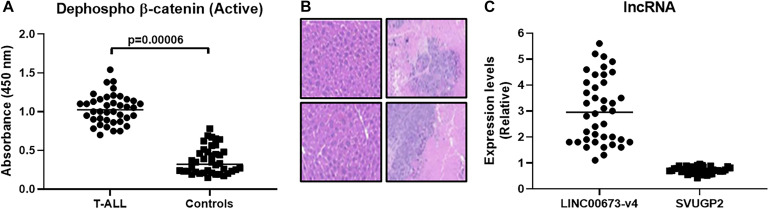
β-catenin expression is elevated in T-ALL patients and favors hepatic metastasis. **(A)** β-catenin expression (active dephosphorylated form) was evaluated, using ELISA, in T-ALL patients (*n* = 40) and compared with that in healthy volunteers (*n* = 40). **(B)** Flow sorting was performed to segregate β-catenin-positive and β-catenin-negative fractions which, after culture, were injected (50,000) into the tail veins of mice (*n* = 6, each), intra-venously. Mice were euthanized after 7 weeks and hepatic metastases evaluated. Representative immunohistochemistry images are shown (Left top and bottom: β-catenin-negative and Right top and bottom: β-catenin-positive). **(C)** lncRNAs (LINC00673-v4 and SVUGP2) were evaluated for their expression in β-catenin-positive and β-catenin-negative fractions and the expression levels in β-catenin-positive, relative to β-catenin-negative fractions, are shown.

With the information that wnt signaling, in particular, β-catenin is over-expressed in T-ALL patients, we first sought to verify this in our patient cohort of 40 patients diagnosed with T-ALL. We performed ELISA in patient-derived samples for quantitation of active β-catenin and compared the levels of β-catenin in patients with corresponding levels in 40 healthy controls. The levels were found to be significantly (*p* < 0.0001) elevated in T-ALL patients, compared to controls (Figure 1A). Almost all healthy volunteers, except for one, had lower β-catenin levels than the T-ALL patients (Supplementary Figure 1). We next confirmed the stem cell-like properties endowed by higher expression of β-catenin. Patient-derived cells (with the highest observed β-catenin levels) were subjected to flow cytometric sorting of single cell suspensions and divided into β-catenin-positive and β-catenin-negative fractions. Both fractions of cells were cultured in the lab until the desired number of cells were obtained. Based on our initial titrations, we injected 50,000 cells into the tail veins of NSG mice and euthanized mice after 7 weeks. The livers were visually inspected for signs of metastases. We found hepatic metastases in all the mice injected with β-catenin-positive cells, whereas little to no hepatic metastasis was evident in mice injected with β-catenin-negative cells, as revealed by IHC (Figure 1B). In light of the reports on the effects of lncRNAs on wnt- β-catenin signaling, we evaluated the levels of lncRNAs LINC00673-v4 and SVUGP2, both of which have been implicated in the regulation of this signaling pathway (Guan et al., 2019; Wei et al., 2019; Lin et al., 2020). We found elevated levels of lncRNA LINC00673-v4 in β-catenin-positive fractions (∼3.02 folds; Figure 1C) which is in agreement with the reported literature that this lncRNA promotes cancer aggressiveness via wnt- β-catenin signaling (Guan et al., 2019). lncRNA SVUGP2 was significantly downregulated in β-catenin-positive fractions (∼0.72 folds; Figure 1C) which supports its reported repression in cancers through involvement of wnt- β-catenin signaling (Wei et al., 2019).

### β-Catenin-Positive Fraction Has Stem Cell Like Properties—Increased Drug Resistance and 3-Dimensional Cell Growth

**FIGURE 2:**
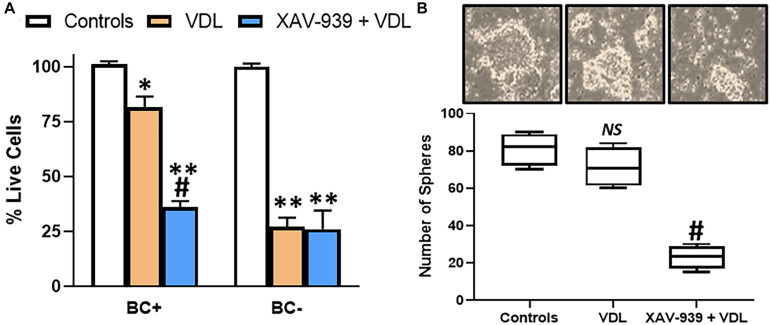
β-catenin confers drug resistance and 3-dimensional cell growth. **(A)** Flow sorted β-catenin-positive (BC+) and β-catenin-negative (BC–) cells were subjected to VDL treatment, as described in section “Materials and Methods,” in the presence and absence of 5μM XAV-939 for 10 days. Live cells were estimated by trypan blue exclusion method. **(B)** Flow sorted β-catenin-positive cells were also allowed to grow in 3-dimensional cultures to generate tumorospheres, in the presence of VDL ± 5μM XAV-939. Representative images of tumorospheres are provided along with the bars representing the number of tumorospheres. ^∗^*p* < 0.05 and ^∗∗^*p* < 0.01, compared to control, *NS*, non-significant, compared to control, ^#^*p* < 0.01, compared to VDL.

Increased metastasis, as observed above, is one hallmark of cancer stem cells. Other hallmarks include increased resistance to therapy and 3-dimensional sphere formation. To study resistance to therapy, we subjected both β-catenin-positive and negative cells to VDL treatment for 10 days. Consistent with the role of β-catenin in stemness, the β-catenin-positive cells were highly resistant to VDL, compared to β-catenin-negative cells, as determined by the number of live cells with and without VDL treatment (Figure 2A). Further, pharmacological inhibition of β-catenin, by XAV-939, resulted in sensitization of resistant cells to VDL (Figure 2A), thus, verifying the essential role of β-catenin in determining resistance to therapy. The concentration of XAV-939 was chosen based on the published work at which it is not cytotoxic to cells but still a potent β-catenin inhibitor (Stakheev et al., 2019). When we generated tumorospheres from β-catenin-positive cells, similar results were observed i.e., VDL had minimal effect on the ability of β-catenin-positive cells to form spheres while pharmacological inhibition of β-catenin significantly decreased the ability to form such spheres (Figure 2B).

### Factors Governing Progression to Tolerance of Inhibition of β-Catenin

**FIGURE 3:**
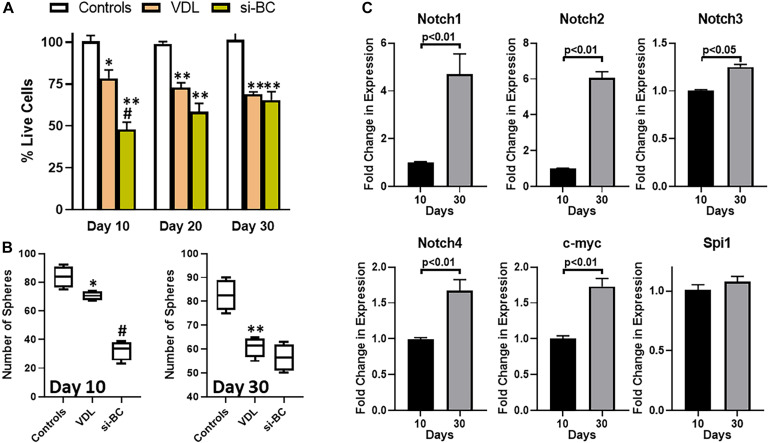
Acquired drug resistance model and alternate signaling. **(A)** Flow sorted β-catenin-positive were subjected to VDL treatment, as described in section “Materials and Methods,” in the presence and absence of si-RNA specific for β-catenin. Live cells were estimated at days 10, 20, and 30, by trypan blue exclusion method. **(B)** Flow sorted β-catenin-positive cells were also allowed to grow in 3-dimensional cultures to generate tumorospheres, in the presence of VDL ± si-RNA specific for β-catenin. Bars represent the number of tumorospheres. **(C)** Gene expression levels of Notch 1–4, c-myc and Spi1, at days 10 vs. days 30 in flow sorted β-catenin-positive cells, while being treated with VDL, were determined by quantitative RT-PCR. Expression of GAPDH was used as internal control. ^∗^*p* < 0.05 and ^∗∗^*p* < 0.01, compared to control, ^#^*p* < 0.01, compared to VDL.

In clinics, T-ALL patients often stop responding to therapy, resulting in disease relapse. We observed sensitization of T-ALL patient derived cells to VDL when β-catenin was pharmacologically inhibited. We asked if prolonged inhibition of β-catenin could result in similar attenuation of sensitization to therapy, and, therefore, we silenced β-catenin by using si RNA against β-catenin. We noticed that passage of time resulted in decreased sensitizing effect of β-catenin-silencing i.e., whereas silencing of β-catenin significantly sensitized T-ALL patient-derived cells to VDL initially (at 10 days VDL treatment), the sensitization was totally lost at 30 days (Figure 3A). Clearly, the cells were now resistant to β-catenin-silencing. The results were verified with sphere assay as well (Figure 3B). At this point, we were interested in understanding the mechanism of this acquired resistance. Based on the available knowledge, we screened for factors that were most likely to surrogate for β-catenin’s leukemia initiating properties. When we compared 10 days samples (when cells were still responding to β-catenin-inhibition) with 30 days samples (time period at which resistance to β-catenin inhibition was obvious), we found elevated levels of all Notch family receptors as well as c-myc, however, Notch1 and Notch2 clearly stood out as the most differentially expressed factors (Figure 3C).

### β-Catenin, Notch and c-Myc Expression in Progressed T-ALL Patients

**FIGURE 4:**
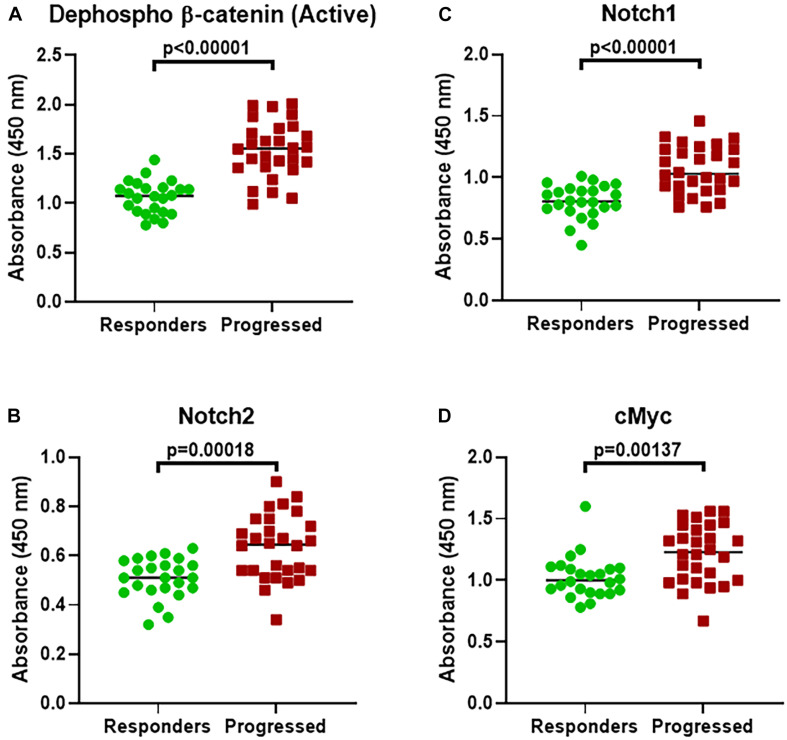
Expression of β-catenin and alternate genes in T-ALL patients. Expression of **(A)** β-catenin (active dephosphorylated form). **(B)** Notch1. **(C)** Notch2. **(D)** c-Myc expression was evaluated, using ELISA, in T-ALL patients who had progressed on their therapy (Progressed, *n* = 28) and compared with those patients who were still responding (Responders, *n* = 24). *P*-values are mentioned for individual genes, as found significant.

To ascertain whether increased β-catenin as well as Notch levels are relevant to T-ALL patients who have acquired resistance and whose disease has relapsed and/or progressed, we recruited T-ALL patients currently undergoing treatment at our Hospital. For this investigation, we identified 24 T-ALL patients (mostly diagnosed within the last 6 months) who were still responding to their assigned threrapies and another 28 patients whose disease had progressed and these patients had stopped responding to their last assigned therapy. We found β-catenin, Notch1, Notch2 as well as c-Myc to be significantly over-expressed in patients whose disease had progressed (Figure 4).

**FIGURE 5:**
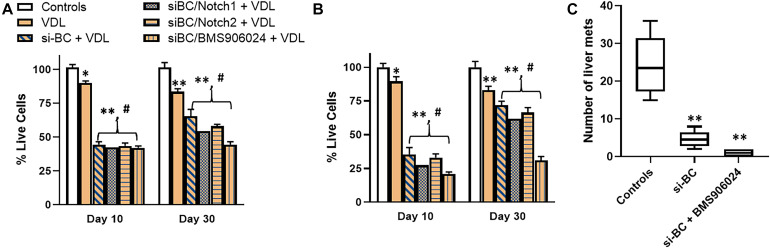
Cells derived from relapsed patients and effect of Notch silencing. β-catenin-high cells derived from **(A)** patient 1 and **(B)** patient 2 were subjected to VDL treatment, as described in section “Materials and Methods,” in the presence and absence of si-RNAs specific for β-catenin (BC), Notch1, Notch2 or 100 nM pan-Notch inhibitor, BMS906024. Live cells were estimated at days 10 and 30, by trypan blue exclusion method. **(C)** Cells (50,000) from patient 2, ± si-β-catenin or si-β-catenin + BMS906024, as indicated, were also injected into the tail veins of mice (*n* = 6, each), intra-venously. Mice were euthanized after 7 weeks and hepatic metastases evaluated. Bar graphs represent number of metastatic nodules counted. ^∗^*p* < 0.05 and ^∗∗^*p* < 0.01, compared to control, ^#^*p* < 0.01, compared to VDL.

Next, we identified 2 patients with progressed disease and the highest expression of β-catenin. Tumor cells were isolated, single cell suspensions made and the cells cultured in the lab. We now wished to study the inter relationship between wnt and Notch signaling, with regards to the maintenance of stemness. In assays performed on cells from one patient, VDL resistance was observed as expected (because of increased β-catenin) (Figure 5A). Further, even though silencing of β-catenin was initially found to sensitize cells to VDL, prolonged treatment of 30 days resulted in significant de-sensitization. We speculated that Notch signaling might be mechanistically responsible and therefore we silenced Notch1 as well as Notch 2, using specific siRNAs. Though some re-sensitization was evident, almost a complete reversal of resistance to β-catenin inhibition was achieved only when a pan-Notch inhibitor (BMS906024) was used. BMS906024, at a concentration of 100nM used here, can inhibit Notch signaling (Morgan et al., 2017). The results were further conformed using cells derived from the second such patient (Figure 5B). The observations in cells derived from this patient were even more significant, which prompted us to further test these cells *in vivo* in a mice metastasis model. Cells from control condition, si-β-catenin and si-β-catenin + BMS906024 were injected in tail veins of mice and as seen in Figure 5C, whereas control cells metastasized to liver, silencing of β-catenin had an inhibitor action. It is possible that the presence of few metastatic hepatic nodules in β-catenin-silenced group might be reminiscent of relapsing disease, which is further supported by the observation that pan-notch inhibitor + β-catenin silencing almost completely blocked the metastasis.

## Discussion

Even though wnt signaling is often de-regulated in hematological malignancies (Polakis, 2012), its role in etiology of T-ALL is not very well understood. In one of the first studies on the subject (Guo et al., 2007), it was reported that β-catenin stabilization predisposes thymocytes to malignant transformation. β-catenin is an essential factor in the canonical wnt signaling pathway wherein activated wnt signaling and binding of wnt ligand to its cognitive receptor leads to accumulation of β-catenin in cytoplasm followed by its translocation to the nucleus where it functions as a transcription factor.

The relevance of Notch signaling in T-ALL is well regarded (Sanchez-Martin and Ferrando, 2017). A role of Notch signaling in T-ALL in mouse (Aster et al., 2000; Feldman et al., 2000; Hoemann et al., 2000) as well as human models (Weng et al., 2003, 2004) has been investigated. It was suggested that more than 50% T-ALLs have activating mutations that involve the extracellular heterodimerization domain and/or the C-terminal PEST domain of Notch1, thus advocating the need for targeted therapies against Notch signaling T-ALL (Weng et al., 2004). More recent data suggests that even higher number of T-ALLs, upto 75%, have activating *Notch1* mutations (Liu et al., 2017). Not just Notch1, but activating mutations in Notch3 have also been implicated in T-ALL (Bernasconi-Elias et al., 2016).

In the report on role of stabilized β-catenin in development of T-ALL, up-regulation of c-Myc was reported as a consistent secondary event (Guo et al., 2007). Moreover, it was noted that such stabilization of β-catenin does not lead to activation of Notch receptors, thus suggesting that wnt signaling-mediated T-ALL might represent T-ALLs that do not depend on Notch signaling. Wnt and Notch signaling, therefore, seemed to be two independent pathways responsible for onset of T-ALLs. Stabilization of β-catenin is perhaps most relevant to childhood T-ALL as more than 85% childhood T-ALL patients, of a total of 71 patients comprising of 53 boys and 18 girls, were reported to express up-regulated β-catenin (Ng et al., 2014). It is important to note that up-regulation of β-catenin was reported to be independent of Notch activation (Ng et al., 2014). While our study does not challenge such notion, we provide evidence in support of a novel regulatory and inter-dependence of wnt and notch signaling pathways. We show that in T-ALLs where β-catenin is activated, its silencing is effective. However, similar to observations in clinics, the therapy is not good forever and eventually T-ALLs become refractory to β-catenin inhibition. It is at this moment that Notch signaling probably takes over as the alternate pathway. This is evident in the multifold increase in expression of Notch receptors, especially Notch 1 and 2. As a sign of further complex regulation, it looks like there is some level of redundancy in this activation of alternate pathway. We report that pan-notch inhibition, as opposed to inhibition of individual Notch receptors is the most effective strategy. This makes it apparent that both Notch 1 and Notch 2 seem to be ready to drive T-ALL growth in case the other Notch and β-catenin is inhibited.

Based on our observations, we propose that future investigations should evaluate simultaneous inhibition of multiple signaling pathways, particularly those that are closely related. Even though we investigated Notch signaling as an alternate pathway when β-catenin was inhibited because of its relevance to T-ALL and the probability of taking up almost all the functions, particularly those connected to stemness, our unpublished preliminary results from high throughput assays indicate that Notch family members indeed are among the top potential candidates that can control the proliferation and metastasis of T-ALL cells when β-catenin inhibition is rendered ineffective.

A number of drugs that target either Notch signaling or β-catenin are progressing through the clinical trials (Ran et al., 2017; Savvidou et al., 2017; Wu et al., 2017; Krishnamurthy and Kurzrock, 2018; Massard et al., 2018). This is an exciting time, however, it cannot be stressed enough that targeting a single signaling molecule/pathway is highly unlikely to yield long term benefits to patients. The future treatment strategy will perhaps include a cocktail of highly effective targeted drugs that will be administered cyclically to help prevent toxicity as well acquisition of resistance. For T-ALL patients, β-catenin as well Notch—targeting drugs will definitely be part of such cocktail.

## Data Availability Statement

The original contributions presented in the study are included in the article/Supplementary Material, further inquiries can be directed to the corresponding author/s.

## Ethics Statement

The studies involving human participants were reviewed and approved by the Institutional Review Board and the Ethics Committee at Sun Yat-sen University. The patients/participants provided their written informed consent to participate in this study. The animal study was reviewed and approved by the Animal Research Ethics Committee at the Sun Yat-sen University.

## Author Contributions

LZ, JW, YF, BK, ZF, and J-JL participated in performing experiments, collecting data, and analyzing results. JG, BT, RX, and GP analyzed data and performed statistical analyses. J-JL conceptualized and supervised study. All authors contributed to the article and approved the submitted version.

## Conflict of Interest

The authors declare that the research was conducted in the absence of any commercial or financial relationships that could be construed as a potential conflict of interest.
