# Supplementary figures and images for: A Regulatory Loop Involving Notch and Wnt Signaling Maintains Leukemia Stem Cells in T-Cell Acute Lymphoblastic Leukemia
Source: Front Cell Dev Biol. 2021 Jun 11;9:678544. doi: 10.3389/fcell.2021.678544 (PMC8226090; doi:10.3389/fcell.2021.678544)

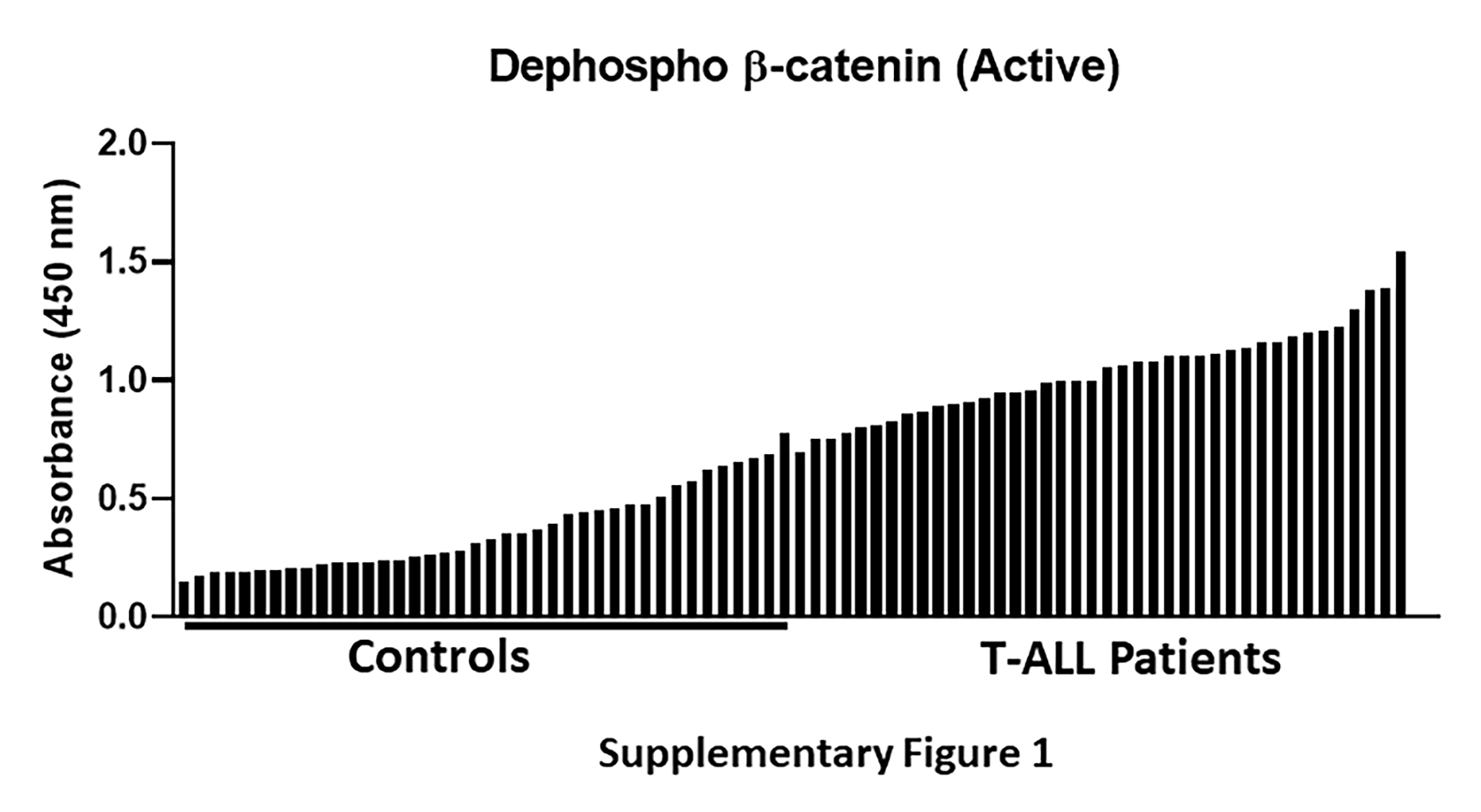

Supplement: Supplementary file 1 [file Image_1.TIF]
